# Fitness costs of resistance to insecticide pymetrozine combined with antimicrobial zhongshengmycin in *Nilaparvata lugens* (Stål)

**DOI:** 10.3389/fphys.2023.1160873

**Published:** 2023-04-13

**Authors:** Xupiaoyang Feng, Danting Li, Hongfeng Wang, Xiaoping Yu, Xuping Shentu

**Affiliations:** Zhejiang Provincial Key Laboratory of Biometrology and Inspection and Quarantine, College of Life Science, China Jiliang University, Hangzhou, China

**Keywords:** *Nilaparvata lugens* (Stål), pymetrozine, zhongshengmycin, fitness costs, two-sex life table

## Abstract

The brown planthopper, *Nilaparvata lugens* (Stål), is a major pest of rice crops, and its control is critical for food security. Pymetrozine has been recommended as an alternative to imidacloprid for controlling *N. lugens*, but the pest has developed high resistance to it, making its prohibition and restriction urgent. To address this issue, we conducted a study using a mixture of pymetrozine and zhongshengmycin with the effective ratio of 1:40, to evaluate the fitness costs in *N. lugens*. Our results showed that *N. lugens* had a relative fitness of 0.03 under this ratio, with significantly reduced longevity, female and male adult periods, total pre-oviposition days, and fecundity. Moreover, the expression levels of the uricase gene (EC1.7.3.3) and farnesyl diphosphate farnesyl transferase gene (EC2.5.1.21) were reduced in *N. lugens*. These genes are involved in urea metabolism and steroid biosynthesis pathway, respectively, and their suppression can interfere with the normal nutritional function of *N. lugens*. Our study demonstrates that the combination of chemical insecticides and antimicrobials can delay the development of resistance and improve the efficiency of pest control. This information is valuable for researchers developing management strategies to delay the development of pymetrozine resistance in *N. lugens.*

## 1 Introduction

The brown planthopper, *Nilaparvata lugens* (Stål), is a monophagous rice pest belonging to the planthopper family and is considered one of the most critical pests in Asia (Bottrell and Schoenly, 2012). *N. lugens* feeds on rice phloem sap (Zhang, 2007), and as a sap-sucking insect, it harbors endosymbionts that are thought to play a vital role in host development (Fan et al., 2015). Due to its monophagy, *N. lugens* harbors obligatory yeast-like symbionts (YLS) that cannot be cultured *in vitro* (Pang et al., 2012). YLS is known to support sterol biosynthesis and nitrogen recycling in the host and are closely related to the life activities of *N. lugens* (Xue et al., 2014).

The impact of *N. lugens* infestation on rice crops in Asia is immeasurable. Chemical pesticides are currently the most commonly used method for *N. lugens* management, but this has led to increasing resistance and serious environmental pollution (Liu et al., 2018). Several studies have reported the development of resistance to a range of insecticides by *N. lugens*, including imidacloprid, buprofezin, thiamethoxam, dinotefuran, clothianidin, isoprocarb, chlorpyrifos, and nitenpyram (Zhang et al., 2014; Bass et al., 2015).

Pymetrozine was first developed in 1988 and released on the market in 1994, it has contact and systemic activities and acts as an agonist of nicotinic acetylcholine receptors (Li et al., 2021). It is highly effective against a wide range of sap-feeding pests by irreversibly blocking the stylets of *N. lugens*, causing them to stop feeding and eventually starve to death. Pymetrozine has a long-lasting effect, but its ability to knock down pests is not strong (He et al., 2011a). While it is one of the recommended alternatives to imidacloprid for controlling *N. lugens* (He, et al., 2011b), its frequent use leads to high levels of resistance in insects, which are likely to continue to increase. Therefore, the use of pymetrozine is now restricted and prohibited. To delay the development of resistance in insects, the use of mixed insecticides is a common method to prolong the service life of insecticides. Pymetrozine mixture products are generally compatible with neonicotinoid insecticides. Research has shown that the maximum co-toxicity coefficient to the third instar nymphs of *N. lugens* can reach 2,813.04 when the compound ratio of pymetrozine and dinotefuran is 2:1, and the synergistic effect of the two mixtures is remarkable (Wang et al., 2013). Additionally, zhongshengmycin, a fungicide, can inhibit symbiotic microorganisms in the fat body of *N. lugens*, thereby increasing the mortality of *N. lugens* (Shi et al., 2021; Song et al., 2021). Thus, we propose a novel method of using a mixture of pymetrozine and a fungicide such as zhongshengmycin to control pests by inhibiting their symbionts.

Insecticide-resistant insects often exhibit increased energy consumption or disturbances in their metabolic balance, resulting in a certain fitness cost (Ullah et al., 2020a). Resistant populations often have slower developmental rates, reduced survival rates and fecundity (Zhang et al., 2018; Liao et al., 2019; Li et al., 2020), these adverse effects of fitness costs on the growth, development, and reproduction of resistant insects are important factors that limit the development of resistance. Therefore, studying the cost of resistance fitness provides theoretical support for formulating resistance control measures (Liao et al., 2019). Studies have also shown that *N. lugens* is at risk of developing resistance to several insecticides, including imidacloprid, fipronil, nitenpyram, chlorpyrifos, and sulfenalazine (Ullah et al., 2020b). However, due to the obvious fitness cost of resistant populations, once selection pressure is lost, the sensitivity of *N. lugens* to an agent can recover quickly after stress. Therefore, when controlling *N. lugens* in the field, the frequency of application of the abovementioned pesticides should be strictly controlled, long-term single use should be avoided, and other types of insecticides without cross-resistance should be used in rotation to effectively delay the development of *N. lugens* resistance (Shi et al., 2011). Fitness costs vary among different species and depend on the types of pesticide treatments and living conditions. Understanding the fitness costs of insecticide resistance can help conduct a more comprehensive evaluation of new pesticide mixtures and design an integrated pest management program.

Previous studies have revealed that the genetic capacities for nitrogen recycling of both *N. lugens* and YLS are complementary (Brentassi et al., 2017), the pathways of the two organisms are highly complementary, with the uricase gene (EC1.7.3.3) being the only shared gene (Xue et al., 2014). The cooperation between the enzymes encoded by the two genomes contributes to the successful conversion of urate into precursors for the biosynthesis of essential amino acids, this nitrogen-recycling pathway serves as an additional source of amino acids for *N. lugens*, indicating that this integrative system is an adaptation *via* host-symbiont co-evolution that enables *N. lugens* to exist solely on a diet of nutritionally limited and unbalanced rice phloem sap. Researchers have also demonstrated that *N. lugens* and YLS have developed an interdependent system for steroid biosynthesis. The ability of YLS to supply sterol may explain how *N. lugens* can survive on a sterol-free diet. In general, the first enzymes of many metabolic pathways are rate-limiting. Therefore, in this experiment, the first gene of the steroid biosynthetic pathway in the YLS genome for the farnesyl diphosphate farnesyl transferase gene (EC2.5.1.21) was selected (Xue et al., 2014; Fan et al., 2015).

In this study, susceptible populations of *N. lugens* without laboratory exposure to chemical pesticides were used to evaluate the fitness costs of pymetrozine, zhongshengmycin, and the pymetrozine–zhongshengmycin mixture. The findings of this study are of great significance for delaying the development of resistance in *N. lugens* and formulating more efficient pest management programs, the results can serve as a reference for establishing an effective control system for *N. lugens*, thereby providing valuable information to researchers in the field.

## 2 Materials and methods

### 2.1 Experimental materials

#### 2.1.1 Insect rearing

The susceptible rice variety TN1 was used in the trials. After soaking for 24 h, seed germination was accelerated for 48 h, and the seeds were evenly spread in an acrylic tray containing perlite. The rice seedlings were grown to three tillers in an artificial climate room and were used to feed *N. lugens*. The test insects were sourced from a rice field in Hangzhou, Zhejiang, China. They were maintained on TN1 in an artificial climate room for more than 10 generations under standard conditions of 26°C ± 1°C, 70%–80% relative humidity (RH), and a 16 h:8 h light/dark photoperiod.

#### 2.1.2 Chemicals

Pymetrozine [(E)-4,5-dihydro-6-methyl-4-(3-pyridylmethyleneamino)-1,2,4-triazin-3(2H)-one] (96.6% active ingredient w/w) was purchased from Jiangsu Weunite Fine Co., Ltd. Zhongshengmycin [1-N-glucosidine-2-amino-L-lysine-2-deoxygulosamine] (12% active ingredient w/w) was provided by Fujian Kaili Biotechnology Co., Ltd.

### 2.2 Fitness comparison

Four treatment groups were investigated in this experiment: a control treatment group (*N. lugens* population feeding on rice plants treated with 0.1% Triton X-100 water solution), a pymetrozine treatment group (*N. lugens* population feeding on rice plants treated with pymetrozine at LC_50_), a zhongshengmycin treatment group (*N. lugens* population feeding on rice plants treated with zhongshengmycin at LC_50_), and a mixture treatment group (*N. lugens* population feeding on rice plants treated with a 1:40 pymetrozine-zhongshengmycin mixture). Separate life tables were constructed for the *N. lugens* populations in the four treatment groups using the age-stage, two-sex life-table approach (Chi et al., 2020; Liu et al., 2020). Approximately 1000 *N. lugens* adults from each group were transferred into a clean acrylic frame (40 cm length, 30 cm width, 50 cm height) with fresh and healthy rice seedlings for oviposition. *N. lugens* were isolated from other insect sources. After 24 h, all adults were removed, and 100 neonate nymphs were randomly housed. Each test insect was numbered and observed individually in transparent plastic test tubes (2.5 cm diameter, 15 cm height) containing fresh rice seedlings until adulthood. Unmated male and female adults were simultaneously paired in tubes containing two fresh rice seedlings and covered with a fine gauze top. During this process, when paired males died, healthy males from the corresponding treatment group were supplemented in a timely manner until the female died. All test tubes were maintained at 26°C ± 1°C and 70%–80% RH under a 16 h:8 h light/dark photoperiod. The nymphs were considered dead if they were unable to move after a slight push with a fine brush. The number of unhatched eggs on the rice seedlings was counted using a microscope after the newly hatched nymphs were counted. Population characteristics, including the development times of the different stages, longevity, and fecundity (female eggs), were recorded daily to establish the life tables.

### 2.3 Statistical analysis

The life-table data for the *N. lugens* individuals were analyzed using an age-stage, two-sex life-table theory (Qin et al., 2021). Through calculations, the adult pre-oviposition period (APOP), total pre-oviposition period (TPOP), population parameters, and other life-table parameters were obtained. Population parameters include the average generation period (*T*), intrinsic growth rate (*rm*), net reproduction rate (*R*_*0*_), and weekly growth rate (*λ*). The relative fitness was calculated based on the relative fitness (*R*_*f*_) = *R*_*0*_/*R*_*0*_ of the control group (Fujii et al., 2020; Zhang et al., 2018). The age-stage-specific survival rate (*S*_*xj*_), age-specific survival rate (*l*_*x*_), female age-specific fecundity (*f*_*x*_), age-specific fecundity (*m*_*x*_), and age-specific maternity (*l*_*x*_ *m*_*x*_) were plotted using GraphPad Prism 9.

### 2.4 Real-time quantitative PCR analysis

After 72 h, 15 surviving individuals from each treatment group were isolated for RNA extraction and RT-qPCR analysis (Chen et al., 2017). Total RNA from males was extracted using a TaKaRa MiniBEST universal RNA Extraction Kit (TaKaRa, Dalian, China), and the cDNA template for qPCR was synthesized using a Perfect Real-Time PrimeScript™ RT Reagent Kit with a gDNA Eraser (TaKaRa). The quality of the extracted RNA was determined using a NanoDrop 2000 spectrophotometer (Thermo Fisher Scientific, Waltham, MA, United States) and confirmed *via* agarose gel electrophoresis. The qPCR reagent was SYBR^®^ Premix ExTaq™ II (Tli RNaseHPlus) (TaKaRa). The specific primers for EC1733 qPCR were EC1733-F and EC1733-R, and the primers for EC25121 qPCR were EC25121-F and EC25121-R. *β*-actin was used as the qPCR internal reference, with the primers actin-F and actin-R (Supplementary Table S1) (Xue et al., 2014). A 20 µL reaction system was used for qPCR using a StepOnePlus™ real-time PCR System (Applied Biosystems, Carlsbad, CA, United States). Three independent biological replicates and three technical replicates were set up. The relative transcript levels of EC1.7.3.3 and EC2.5.1.21 in different samples were determined using the 2^−ΔΔCT^ method (Maroniche et al., 2011; Zheng et al., 2021).

## 3 Results

### 3.1 Development period comparison

The life history parameters of the *N. lugens* populations in the control group and three treatment groups were summarized (Table 1). Although inconsistent differences were observed between the treatment groups for *N. lugens* in the 1–5 instar nymph stages, the nymph life span in the three treatment groups was longer than that in the control group, with the preadult life span being longest in the mixture treatment group. Among the four groups, the adult period and longevity of *N. lugens* were shortest in the mixture treatment group, and the APOP was significantly longer in the mixture treatment group than in the control and other treatment groups. The number of eggs laid in the mixture treatment group was the lowest, which was significantly lower than that in the control group and the other two treatment groups. These findings suggest that the combination of pymetrozine and zhongshengmycin (1:40) had more detrimental effects on the growth parameters, development status, survival, and reproductive ability of *N. lugens* than either insecticide alone.

**TABLE 1:** Developmental period of *N. lugens* after treatment with different pesticides and mixture.

Stage	Treatment			
	Water mean ± SE^b^	Pymetrozine mean ± SE	Zhongshengmycin mean ± SE	Mixture^a^ mean ± SE
First instar (day)	2.46 ± 0.05a^c^	2.77 ± 0.08ab	2.75 ± 0.10ab	2.90 ± 0.10b
Second instar (day)	2.30 ± 0.05a	2.85 ± 0.07bc	2.60 ± 0.11ab	3.10 ± 0.10c
Third instar (day)	2.73 ± 0.05a	3.81 ± 0.08b	3.45 ± 0.11c	4.10 ± 0.10d
Fourth instar (day)	3.17 ± 0.07a	3.62 ± 0.11a	3.60 ± 0.11a	3.60 ± 0.16a
Fifth instar (day)	3.46 ± 0.07a	2.54 ± 0.11b	2.80 ± 0.11b	3.00 ± 0.15b
Pre-adult (day)	14.12 ± 0.10a	15.58 ± 0.17b	15.20 ± 0.16b	16.70 ± 0.26c
Female adult (day)	15.52 ± 0.58a	8.62 ± 0.45b	6.30 ± 0.68b	6.83 ± 0.79b
Male adult (day)	17.33 ± 0.70a	7.62 ± 0.87b	5.70 ± 0.99b	7.50 ± 0.87b
Longevity (day)	30.55 ± 0.46a	23.69 ± 0.50b	21.20 ± 0.54b	23.80 ± 0.66b
APOP (day)	2.98 ± 0.14a	4.54 ± 0.144b	4.00 ± 0.19ab	4.50 ± 0.29b
TPOP (day)	19.19 ± 0.29a	15.77 ± 0.20b	19.25 ± 0.62a	18.50 ± 0.29a
Fecundity (eggs female)	603.93 ± 14.18a	204.38 ± 8.83b	134.13 ± 13.37bc	74.00 ± 7.04c

e Note: ^a^Mixture tratment: the effective component mass ratio of pymetrozine to zhongshengmycin is 1:40.

e ^b^Data in th table are expressed as the means ± SE, and the numerals in brackets represent the number of repetitions.

^c^ Means followed by different letters within a column are significantly different according to paired bootstrap test (*p* < 0.05).

### 3.2 Population parameter comparison

Population parameters, including *T*, *rm*, *R*_*0*_, and *λ*, were analyzed for each treatment group using TWOSEX MSChart software (Table 2). The *T* (day) of *N. lugens* in the mixture treatment group was 20.05 days, indicating that the population treated with the mixture required the shortest time to complete a generation cycle. Meanwhile, the *rm* (d^−1^) of *N. lugens* in the mixture treatment group was 0.06, indicating that the population growth trend in this group was the slowest among all the groups. The *λ* (d^−1^) of *N. lugens* in the mixture treatment group was 1.062/d, with this population producing the fewest offspring per individual under conditions of unlimited resources. The *R*_*o*_ (d^−1^) of *N. lugens* in the mixture treatment group was 3.34, indicating that this population had the smallest multiplication after one generation cycle. The *R*_*f*_ value for the mixture treatment group was 0.0349, calculated from the *R*_*0*_ value, which was the lowest among the four groups. These results demonstrate that fitness costs were associated with insecticide resistance in *N. lugens*, and that the combination of pymetrozine and zhongshengmycin can effectively inhibit the development, survival, and progeny reproduction of *N. lugens* populations.

**TABLE 2:** Population parameters of *N. lugens* after treatment with different pesticides and mixture.

Parameter	Water	Pymetrozine	Zhongshengmycin	Mixture^a^
*T* (day)	22.74	21.04	21.36	20.05
*rm* (d^−1^)	0.201	0.156	0.125	0.060
*λ* (d^−1^)	1.223/d	1.169/d	1.133/d	1.062/d
*R*_*0*_ (offspring individual^−1^)	95.71 ± 3.59	26.39 ± 1.22	14.51 ± 0.98	3.34 ± 0.89
*R*_*f*_ ^b^		0.276	0.152	0.035

^a^ Note: Mixture treatment: the effective component mass ratio of pymetrozine to zhongshengmycin is 1:40.

^b^ *R*_*f*_ = *R*_*0*_ of the other treatment group/*R*_*0*_ of control treatment group.

### 3.3 Life table curve comparison

**FIGURE 1:**
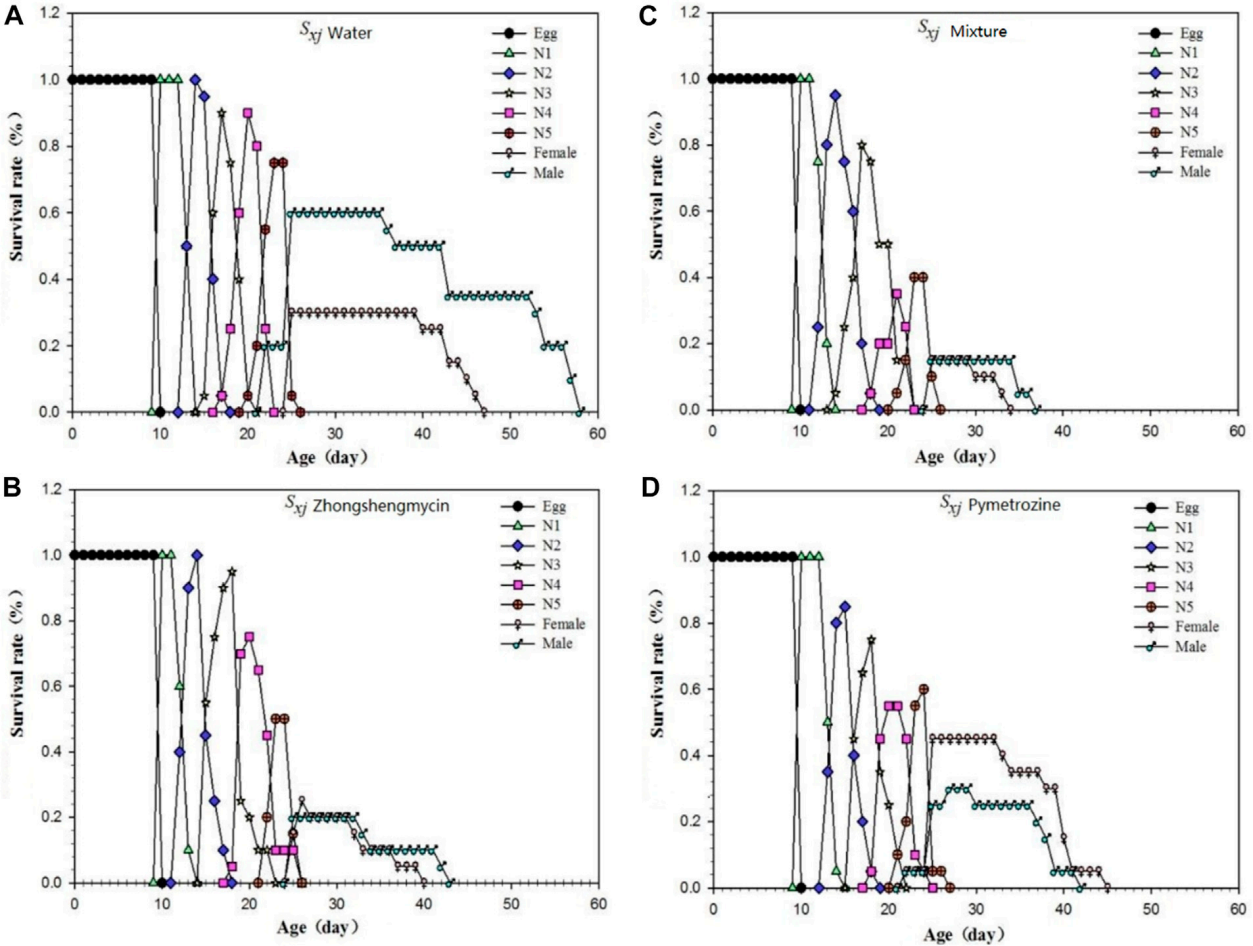
Age-stage-specific survival rate of *N. lugens* **(A)** Age-stage-specific survival rate (*S*_*xj*_) of *N. lugens* in the water treatment; **(B)** age-stage-specific survival rate (*S*_*xj*_) of *N. lugens* in the zhongshengmycin treatment; **(C)** age-stage-specific survival rate (*S*_*xj*_) of *N. lugens* in the mixture treatment; **(D)** age-stage-specific survival rate (*S*_*xj*_) of *N. lugens* in the pymetrozine treatments.

**FIGURE 2:**
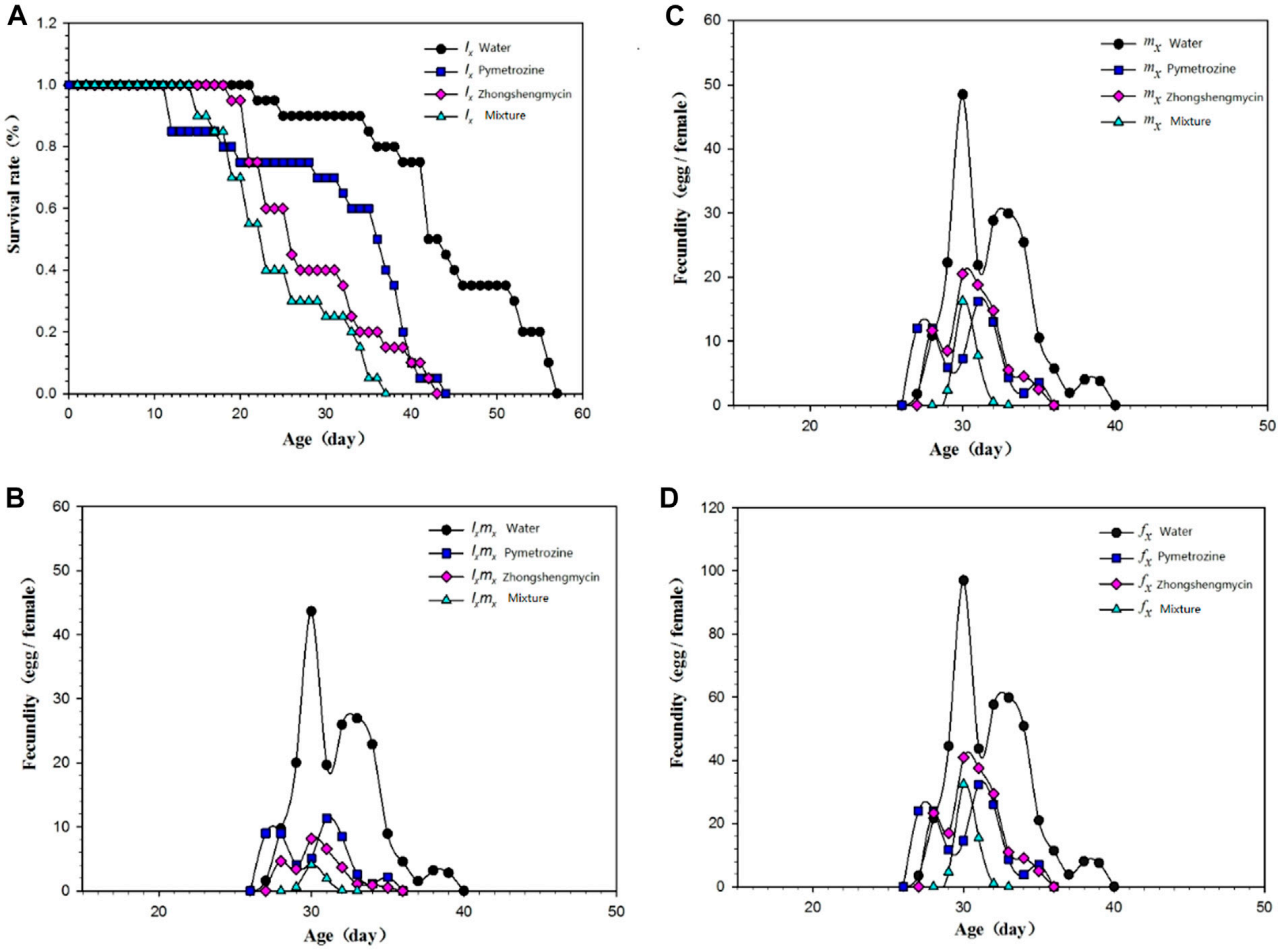
Age-specific survival rate and fecundity of *N. lugens* **(A)** Age-specific survival rate (*l*_*x*_) of *N. lugens* in different treatments; **(B)** age-specific maternity (*l*_*x*_ *m*_*x*_) of *N. lugens* in different treatments; **(C)** age-specific fecundity of *N. lugens* in different treatments (*m*_*x*_); **(D)** female age-specific fecundity (*f*_*x*_) of *N. lugens* in different treatments.

The age-stage survival rate (*S*_*xj*_) represents the probability that a newly laid egg will survive to age *x* and stage *j* (Figure 1). In this experiment, *N. lugens* with the same incubation period were selected for subsequent records to minimize the differences caused by different incubation periods. The curves for the mixture and the zhongshengmycin treatment groups show similar patterns. However, the survival of *N. lugens* in different incubation periods were higher in the zhongshnegmycin treatment group than the mixture treatment group. The age-specific survival rate (*l*_*x*_) without considering the different stages, which is a simplification of *S*_*xj*_, also showed a similar pattern for the mixture and zhongshengmycin treatments (Figure 2A). The *l*_*x*_ value was found to decrease in the late growth stage, which is the period of high mortality for *N. lugens* populations. The *l*_*x*_ of the pymetrozine treatment decreased first, followed by the mixture and zhongshengmycin treatments, and last, by the control, which had a shorter plateau period that delayed the downward trend. These results verify that pymetrozine does not have a strong ability to knock down pests. The overall trends of the *m*_*x*_ (Figure 2C), *l*_*x*_ *m*_*x*_ (Figure 2B), and *f*_*x*_ (Figure 2D) curves initially increase and then decrease. However, in terms of the *m*_*x*_ values, the following trend was observed: water treatment > pymetrozine treatment > zhongshengmycin treatment > mixture treatment (Figure 2C). For the entire observation period, the age-specific fecundity, age-specific maternity, and female age-specific fecundity of *N. lugens* in the mixture treatment group were lower than those of the other three treatment groups. (Figures 2B, D). These results indicate that the mixture treatment had the best control efficiency against *N. lugens*.

### 3.4 Expression of EC1.7.3.3 and EC2.5.1.21 in *N. lugens*

**FIGURE 3:**
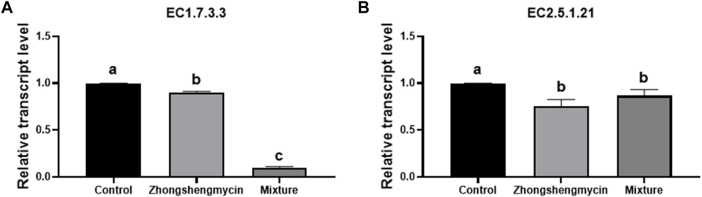
Expression level of EC1.7.3.3 and EC2.5.1.2.1 in *N. lugens* **(A)** Expression of EC1.7.3.3 in *N. lugens* with different treatments, **(B)** expression of EC2.5.1.2.1 in *N. lugens* with different treatments. After 72 h, 15 surviving individuals from each treatment group were isolated for RNA extraction and RT-qPCR analysis. Data are presented as the means ± standard error. Statistical analyses were conducted using one-way ANOVA and unpaired two-tailed Student’s t-test using GraphPad Prism Software 8.0.2 (GraphPad Software, San Diego, CA, United States). Lowercase letters indicate significant differences (*p* < 0.05).

The results indicate that the expression level of the uricase gene (EC1.7.3.3) (gene ID: NLU006642.1) was significantly higher in the control group than in the zhongshengmycin and mixture treatment groups. Notably, the expression level in the mixture treatment group was lower than that for the zhongshengmycin treatment group (Figure 3A). EC1.7.3.3 is involved in both the nitrogen recycling and ammonia assimilation pathways in *N. lugens*, and the pathways would be compromised if either *N. lugens* or its YLS lost just one of the current genes that encode the enzymes involved in these pathways. The addition of zhongshengmycin affects YLS, which indirectly affects the expression of this gene. The mixture treatment not only inhibits YLS but also affects *N. lugens*, leading to a more pronounced effect on the expression of the uricase gene.

The results also show that the expression level of the farnesyl diphosphate farnesyl transferase gene (EC 2.5.1.21) (gene ID: A1835) was lower in the control treatment group than in the zhongshengmycin and mixture treatment groups (Figure 3B). The ability of YLS to supply sterol may explain how *N. lugens* can survive on a sterol-free diet. Thus, the inhibition of YLS by zhongshengmycin as an antimicrobial agent indirectly affected the expression of EC2.5.1.21 in *N. lugens*.

## 4 Discussion

Although pymetrozine has no direct insecticidal activity, it has a unique toxicological mechanism (He et al., 2011b; Yang et al., 2016). Electrical penetration graph evidence showed that pymetrozine toxicity to *N. lugens* occurs through the inhibition of phloem feeding. In addition, pymetrozine interferes with the reproductive behavior of *N. lugens* and hearing in insects (Liu et al., 2018; Wang et al., 2020). Subsequent studies on fruit flies identified the Nan-Iav TRPV channels as the target of pymetrozine and its analog pyrifluquinazon (Wang et al., 2019). The metabolic resistance mechanism of *N. lugens* to pymetrozine involves overexpression of cytochrome P450 *CYP6CS1* (Wang L. et al., 2021). Zhongshengmycin, on the other hand, is a low-toxicity aminoglycoside antibiotic produced by *Streptomyces lavendulae* var and is widely used as a chemical bactericide in the control of phytopathogens. Previous studies have shown that zhongshengmycin can effectively control vegetable bacterial diseases, rice bacterial leaf blight, and fruit tree diseases. Moreover, it has good herbicidal and fungicidal properties, making it a control agent in numerous new mixture studies. In addition, zhongshengmycin has been found to inhibit *Xanthomonas oryzaepv. oryzae*, causing changes in bacterial communities (Wang Q. et al., 2021). While zhongshengmycin is effective in controlling plant pathogens, it can also affect the microbiome in the fat body through feeding behavior, which can influence the mortality of *N. lugens* (Shi et al., 2021).

The present study is the first to investigate the combined effects of pymetrozine and the antimicrobial agent zhongshengmycin on controlling *N. lugens*. The effective ingredient mass ratio of 1:40 pymetrozine and zhongshengmycin showed a significant synergistic effect on controlling *N. lugens*. This effect is likely due to the antimicrobial properties of zhongshengmycin. Furthermore, the relationship between pymetrozine and zhongshengmycin is not just additive or amplifying but may involve complex interactions. The mixture not only improved the control effect of pymetrozine but also reduced the amount of pymetrozine used. The mechanisms of action of pymetrozine and zhongshengmycin are distinct, and the two agents do not exhibit cross-resistance. Typically, pesticide combinations involve different pesticides, different fungicides, or biological and chemical pesticides (He et al., 2013). The combination of an insecticide and an antimicrobial agent used in this experiment is relatively novel. The synergistic effect produced by the combination of agents can lead to a better control effect than using a single agent. This approach expands the insecticidal spectrum and reduces the dosage of the primary agent, thereby allowing the pesticide to exert its maximum effect at the lowest dosage.

Insecticide resistance often comes with fitness costs, which have been observed in many insect pests, including *Bradysia odoriphaga*, *Thrips hawaiiensis*, *Plutella xylostella*, *N. lugens*, and *Musca domestica*, and are considered a crucial in the evolution of resistance (Ullah et al., 2020a). Fitness costs accompanied by high energy costs or other significant disadvantages are generally observed during the development of pesticide resistance (Kliot and Ghanim, 2012). Our study on fitness costs shows that after treatment with insecticides, the pre-adult period of *N. lugens* was prolonged, the adult period and lifespan were shortened, and the survival rate and fecundity levels decreased. Understanding the age differentiation in the population and the differences in survival rates and fecundity at different stages can help improve of biological control of pests. Population parameters, including *T*, *rm*, *R*_*0*_, and *λ*, significantly decreased after treatment with the pymetrozine-zhongshengmycin mixture, indicating that it effectively inhibits the growth rate of *N. lugens*. The mixture showed greater control of *N. lugens* populations than pymetrozine and zhongshengmycin alone, which is consistent with the shorter lifespan and lower fecundity was observed in insecticide-resistant insects screened for sulfoxaflor or nitenpyram resistance (Liao et al., 2019). To cope with the toxicity of insecticides, organisms require sufficient energy and resources for adaptation and survival. The fitness costs of insecticide resistance are considered an important factor limiting the evolution of resistance. These findings can be useful in developing effective resistance management strategies.

*N. lugens* has been shown to have a close symbiotic relationship with endosymbionts. Recent studies have shown that the ability to migrate and resistance to external environmental conditions are related to the abundance and variability of endosymbionts. In particular, *N. lugens* provides a habitat for endosymbionts, which in turn, contribute to its nutrition, growth, development, and ability to adapt to the environment. YLS is one of these endosymbiotic bacteria; it can synthesize essential amino acids, steroids, and vitamins to ensure the nutritional supply of *N. lugens* and the normal functioning of a number of important physiological functions (Vigneron et al., 2012; Laughton et al., 2016; Horgan et al., 2021). Thus, controlling *N. lugens* infection on rice by inhibiting symbionts using antimicrobials is feasible. There are complementary metabolic pathways between *N. lugens* and its symbionts, among which the uricase gene is a typical deletion gene involved in the nitrogen cycle and ammonia assimilation metabolic pathway. The uricase gene (EC 1.7.3.3) is present in the YLS and *N. lugens* genomes, and contributes to the conversion of uric acid to form the precursor of essential amino acid biosynthesis. It provides essential nutrients for the survival of *N. lugens* which are monophagous. Among the genes involved in the steroid biosynthesis pathway, the farnesyl diphosphate farnesyl transferase gene (EC 2.5.1.21) exists in the YLS genome, which converts farnesyl pyrophosphate into squalene in the branch chain of terpenoid biosynthesis, and is a key gene in sterol biosynthesis. To provide sterol nutrients necessary for the survival of *a* single diet of *N. lugens*. The metabolic genome verification of *N. lugens* and its symbionts showed that they were complementary. The complementarity of nutritional pathways provided a theoretical basis for understanding the interactions among *N. lugens* and its symbionts, and emphasized the potential direction for effective control of *N. lugens*. After being treated with zhongshengmycin, the relative expression level of EC 1.7.3.3 (geneID:NLU006642.1), which is involved in nitrogen cycling and the ammonia assimilation pathway was significantly decreased compared with the control group, while the relative expression level of EC 2.5.1.21 (geneID: A1835), which is involved in the steroid biosynthesis pathway, was extremely significantly decreased. The metabolic genome verified that *N. lugens* and YLS are highly complementary, and EC 1.7.3.3 is the only shared gene between *N. lugens* and YLS, which plays an important role in the urea metabolism pathway. At the same time, there is an interdependent steroid biosynthesis system between *N. lugens* and YLS, and EC 2.5.1.21 is only in YLS, which is closely related to sterol and sterol synthesis. Therefore, we can speculate that the decreased gene expression may be due to the inhibition of zhongshengmycin on the *N. lugens* and YLS genes, affecting the genes encoding related enzymes in this biological pathway and destroying the coevolutionary reciprocal relationship between *N. lugens* and YLS.

It is important to note that laboratory experiments may not fully reflect field conditions. Variations in the target population, differences in the degree of anti-drug resistance, and experimental errors can all result in different outcomes. Non-etheless, these results can serve as a useful early warning for pest managers and have practical implications for assessing the efficacy of new pesticides. However, it is necessary to verify the actual control effect of the mixture in the field through field efficacy tests. The relationship between the development of resistance and the cost of fitness can be a foundation for the development of new pesticides. Our study provides valuable insights for researchers to develop effective management strategies for delaying the development of pymetrozine resistance and establishing a sustainable control system for *N. lugens*.

## 5 Conclusion

This study demonstrates that combining pymetrozine with zhongshengmycin enhances the insecticidal effect of pymetrozine and its quick-acting properties. Additionally, the results indicate that antibiotics can impact YLS by inhibiting the expression of genes involved in critical pathways. These findings deepen our understanding of the impact of insect symbionts on insect life activities. Given that field populations of *N. lugens* have shown increasing resistance to pymetrozine, it is crucial to use pymetrozine prudently. In light of this resistance, a cautious approach should be taken when using pymetrozine. Furthermore, considering the fitness cost of pests, developing more mixtures could reduce the use of pymetrozine while improving control effectiveness.

## Data Availability

The original contributions presented in the study are included in the article/Supplementary Material, further inquiries can be directed to the corresponding authors.
