# Supplementary material for: Fitness costs of resistance to insecticide pymetrozine combined with antimicrobial zhongshengmycin in Nilaparvata lugens (Stål)
Source: Front Physiol. 2023 Apr 13;14:1160873. doi: 10.3389/fphys.2023.1160873 (PMC10133562; doi:10.3389/fphys.2023.1160873)
Supplement: Supplementary file 1 [file Table1.docx]

**Supplementary Table S1** The primers used in this study

| Primers | Primer Sequence (5′–3′) |
| --- | --- |
| β-Actin-F | GATGAGGCGCAGTCAAAGAG |
| β-Actin-R | GTCATCTTOTCACGGTTGGC |
| Noda-F | CGTAGGAGAGCAGCAAAC |
| Noda-R | CGATGCCAGAGCCAAGA |
| EC1.7.3.3-F | ACCGTGCATACAACGAAGCA |
| EC1.7.3.3-R | GTGGCTTGTACCGATGGAGA |
| EC2.5.1.21-F | ACCAGCATCTCAATCGCCAG |
| EC2.5.1.21R | CAATGACCGCCGCAAATGAT |
